# Changes in the Cath Lab in the Treatment of Adult Patients with Congenital Heart Disease: A 12-Year Experience in a Single Referral Center with the Establishment of a Dedicated Working Group

**DOI:** 10.3390/jcdd10080314

**Published:** 2023-07-25

**Authors:** Maria Giulia Gagliardi, Roberto Formigari, Marco Alfonso Perrone, Elettra Pomiato, Francesca Fanisio, Mario Panebianco, Rosaria Barracano, Paolo Guccione, Rosalinda Palmieri, Massimiliano Raponi, Lorenzo Galletti

**Affiliations:** 1 Department of Cardiac Surgery, Cardiology and Heart Lung Transplant, Bambino Gesù Children’s Hospital IRCCS, 00165 Rome, Italy; roberto.formigari@opbg.net (R.F.); elettra.pomiato@gmail.com (E.P.); fanisio.francesca@gmail.com (F.F.); mario.panebianco@opbg.net (M.P.); rosaria.barracano@ospedalideicolli.it (R.B.); paolo.guccione@opbg.net (P.G.); rosalinda.palmieri@opbg.net (R.P.); lorenzo.galletti@opbg.net (L.G.); 2 Division of Cardiology and Cardio Lab, Department of Clinical Sciences and Translational Medicine, University of Rome Tor Vergata, 00133 Rome, Italy; 3 Medical Directorate, Bambino Gesù Children’s Hospital IRCCS, 00165 Rome, Italy; massimiliano.raponi@opbg.net

**Keywords:** adult congenital heart disease, interventional cardiology, cardiac catheterization, ACHD

## Abstract

Background: Adults with congenital heart disease (ACHD) are a growing population needing ongoing care. The aim of this study was to investigate if a dedicated ACHD team impacted the timing and indication of invasive cardiology procedures in these patients at our hospital. Methods: Our retrospective single-center study enrolled adult patients with moderate or complex congenital heart disease and with at least one cardiac catheterization between January 2010 and December 2021. According to the period, procedures were labeled as group A (2010 to 2015) or group B (2016 to 2021) and further divided into diagnostic (DCC) and interventional cardiac catheterizations (ICC). Results: 594 patients were eligible for the study. Both DCC (*p* < 0.05) and ICC increased between groups A and B (*p* < 0.05). In group B: Fontan patients accounted for the majority of DCC (*p* < 0.001), while DCC decreased in arterial switch repair (*p* < 0.001). In Fontan patients, conduit stenting was prevalent (*p* < 0.001), while fenestration closures dropped (*p* < 0.01). In patients with tetralogy of Fallot and native outflow tract, percutaneous pulmonary valve implantations (PPVI) increased, with a concurrent reduction in pulmonary valve replacements (*p* < 0.001 vs. surgical series). In right ventricular conduits, ICC increased (*p* < 0.01), mainly due to PPVI. Among Mustard/Senning patients, baffle stenting increased from Group A to Group B (*p* < 0.001). In patients with pulmonary atresia and biventricular repair, ICC often increased for pulmonary artery stenting. Conclusions: A dedicated working group could improve ACHD patients’ indications for interventional procedures, leading to tailored treatment, better risk stratification and optimizing time until heart transplantation.

## 1. Background

The management of patients with congenital heart disease (CHD) has significantly improved over the past few decades; about 90% of children born with CHD now reach adulthood [1]. At present, adult patients with CHD (ACHD) account for two-thirds of the total number of patients [2], but this new population often face chronic and complex sequalae after lifesaving surgery [3] and require a dedicated medical team [4]. In the past, these patients were followed up by pediatric cardiologists, but over the years, clinical and scientific evidence has shown that, to best treat these special cases, a dedicated and multidisciplinary team with expertise in both pediatric and adult cardiology is needed [4].

In this setting, the role of cardiac catheterization procedures in patients with CHD has rapidly evolved from a diagnostic tool to a therapeutic alternative to cardiac surgery [5].

In fact, for some subsets of patients, cardiac MRI and CT studies have focused on diagnostic catheterizations, at same time providing more anatomical and functional information. Furthermore, it is well known from adult populations with valvular diseases that percutaneous treatments are increasingly becoming more widespread compared to cardiac surgery, particularly in high-risk patients, reducing mortality and morbidity [6]. More recently, the same thing happened in ACHD [7] as late post-surgical sequelae are responsive to percutaneous treatment. In particular, new scenarios have also been opened by the use of the percutaneous valves in the right ventricle outflow tract, with the new self-expanding valves prompting an effective alternative to surgery even in the most challenging anatomies. Among complex ACHD, the group of patients with univentricular circulation comprise a relatively heterogenous group of different defects with the common trait of the unique hemodynamic of Fontan circulation [8]. This group of patients in particular requires a dedicated program, a multidisciplinary approach and an aggressive motivation to reach the best possible hemodynamic condition [8].

However, since ACHD patients are an emerging population, data are limited, and all major available studies have some kind of methodological flaw. Previous studies were based on national registries, thus including non-specialized tertiary centers or pediatric and adult patients, or the study population primarily comprised of patients with mild congenital heart disease [7,9,10,11,12,13].

In 2016, the Bambino Gesù Children’s Hospital, a tertiary Italian national reference center for pediatric cardiology and cardiac surgery, started a dedicated program for ACHD patients. The ACHD team comprised cardiologists experienced in both pediatric and adult cardiology and proficient in all non-invasive cardiology testing, multimodality imaging, clinical evaluation, arrhythmias management, and performing cardiac catheterization procedures. Before 2016, ACHD patients were followed up by a general pediatric cardiac outpatient clinic or by the cardiologist who had taken care of them since birth or childhood.

Due to this shift in ACHD care strategy, the aim of this study was to evaluate if the institution of a specialized team may have had an impact on the selection criteria, overall management and prognosis in a cohort of ACHD patients who were candidates for invasive cardiological diagnostic or interventional procedures. This was achieved by comparing the data from cardiac catheterizations carried out within two different time frames: 6 years prior to and 6 years after the start of the ACHD program.

## 2. Methods

This is a retrospective, observational, single-center study on patients followed up from 2010 to 2021 at the Bambino Gesù Children’s Hospital IRCCS, aged ≥18 years old at the time of cardiac catheterization and affected by moderate or complex CHD according to the 2020 Classification of European Society of Cardiology (ESC) [4]. Simple CHD such as atrial septal defects (ASD), ventricular septal defects (VSD), mild pulmonary stenosis, isolated congenital mitral or aortic valve disease, small or repaired ASD, VSD, or patent ductus arteriosus were excluded.

Data were collected from the cardiac catheterization laboratory registry of our Hospital.

Procedures were divided into two main groups: those performed within 6 years before (2010–2015, group A) and those performed 6 years after (2016–2021, group B) the start of the ACHD program, which began on 1 April 2016. The two main groups were further divided up as diagnostic cardiac catheterizations (DCCs) and interventional cardiac catheterizations (ICCs). In all groups, the following variables were examined: age at the time of the procedure, anatomical/functional diagnosis type and final outcome of the procedure.

The anatomical/functional diagnosis types were: univentricular CHD palliated with Fontan circulation, repaired tetralogy of Fallot (rToF) and native outflow tract, transposition of great arteries (TGA) after atrial or arterial switch, biventricular CHDs with right ventricle-to-pulmonary arteries (RV-PA) conduit (rToF, TGA after Rastelli operation, pulmonary atresia with ventricular septal defect, Ross procedure for aortic valve disease), native or operated aortic coarctation (CoAo) and pulmonary atresia with intact ventricular septum (PA/IVS) after biventricular repair.

The study was approved by the Ethics Committee of Bambino Gesù Children’s Hospital IRCCS (protocol code 18/2023) and all subjects signed an informed consent form at the time of hospitalization. The study was conducted in accordance with the Declaration of Helsinki.

## 3. Statistical Analysis

Statistical analysis was performed using SPSS 20.0 software (IBM Corporation, Ar-monk, NY, USA). Continuous variables are presented as mean ± standard deviation (SD), and nominal variables are presented as absolute and relative frequency. Differences between the two groups were tested according to Chi-square test or Fisher’s exact test for nominal variables and with Student’s *t*-test for continuous variables. *p*-values < 0.05 were considered significant.

## 4. Results

Out of 2600 ACHD patients followed up at our center, 1671 (64.2%) had moderate or complex CHD. Among these, 594 had at least one diagnostic or interventional procedure in the catheterization laboratory between 2010 and 2021 and were included in the current study.

Demographic and clinical differences between group A and group B patients are shown in Table 1.

Because some patients had more than one procedure within the time frame of the study, we sought the number and type of procedures, instead of the raw number of patients, as a more accurate means to describe the workflow of an ACHD unit in the catheterization laboratory.

Table 2 shows the raw data and descriptive statistics of the procedures in relation to the type of native or post-surgical condition.

### 4.1. Overall Catheterization Laboratory Workload

From January 2010 to December 2021, 841 procedures were carried out on 594 patients, of which 504 were diagnostic and 337 were interventional.

**Figure 1:**
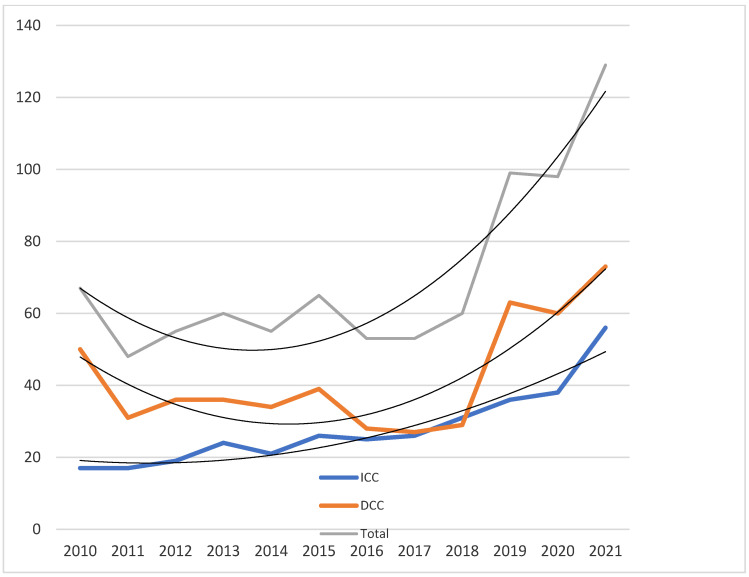
Evolving numbers of diagnostic (DCC), interventional (ICC), and total invasive procedures with polynomial trend curves.

The raw data show an overall increase in the number of procedures over time, with a significant difference between groups A and B (Table 2) for both the diagnostic as well as the interventional groups, with a sharp increase in the number of procedures after 2015, with a steady increase over time (Figure 1). The linear regression analysis shows a statistically different slope of the curves, starting from 2016 (*p* < 0.05 for ICC and *p* < 0.01 for DCC). Interestingly, the number of DCC procedures showed a much steeper increase over time, mostly due to a change in the institutional policy of the invasive monitoring of Fontan patients.

### 4.2. Fontan Circulation

**Figure 2:**
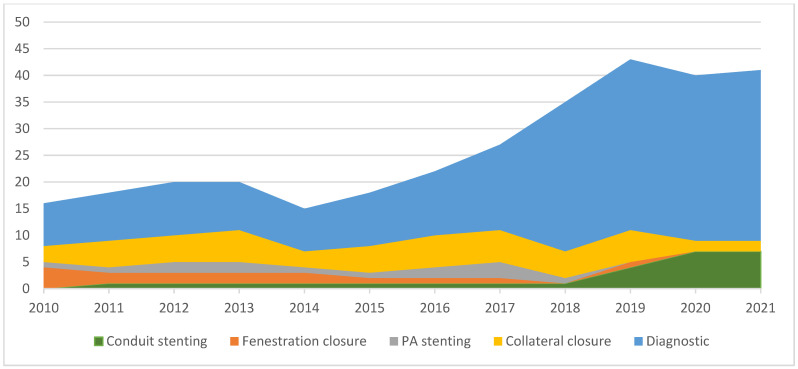
Evolving procedures in adult patients with Fontan circulation.

Among the patients with Fontan circulation, 213 had some type of cardiac catheterization done over the last 12 years. There was a significant increase in the overall number of performed procedures, mostly diagnostic rather than interventional (Figure 2). The increase in diagnostic procedures is due to the adoption of a more aggressive follow-up protocol of Fontan patients, according to the contemporary guidelines. When looking at the different types of interventional catheterization, an increase was noted in Fontan conduit stenting interventions (3 in group A vs. 21 in group B, *p* < 0.001), reflecting the increasing awareness of the need for Fontan circuit optimization and the tendency of progressive conduit stenosis over time. The percutaneous closure of Fontan fenestrations caused a dramatic decrease in the number of procedures from 10 in group A to 3 in group B (*p* < 0.01). This was due to the institutional adoption of more restrictive criteria for fenestration closure by the ACHD team, as opposed to the strategy of previous years of setting up a dedicated working group. There were no differences between the two groups concerning procedures for the embolization of collaterals (25 vs. 32 for group A and B, respectively, *p* > 0.05) or stent/angioplasty of pulmonary arteries (8 vs. 10 in groups A and B, respectively, *p* > 0.05).

### 4.3. Repaired Tetralogy of Fallot with Native Outflow Tract

**Figure 3:**
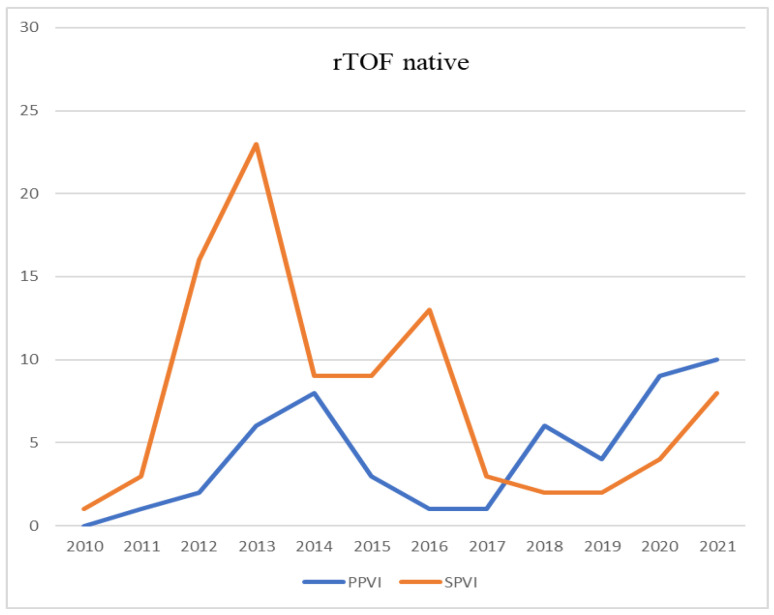
Trends in percutaneous (PPVI) and surgical (SPVI) pulmonary valve implantation in patients with tetralogy of Fallot with native right ventricular outflow tract.

While diagnostic procedures did not increase over time in rToF patients, the number of interventional catheterizations shows a significant increase. Among these patients, percutaneous pulmonary valve implantation (PPVI) was the most frequent type of intervention increasing from 20 in group A to 31 in group B, but without reaching statistical significance. This may be explained by the fact that the basic criteria for valvular implantation in chronic pulmonary regurgitation did not significantly differ over time and were well known among clinical and interventional cardiologists. Interestingly, there was a mutual reduction in surgical pulmonary valve interventions (SPVI) between the 2010–2015 and 2016–2021 time frames (61 vs. 32, *p* < 0.001). For the same reason, the prevalence of pulmonary artery stenting did not differ between the two groups (8 vs. 11, *p* > 0.05). Figure 3 shows the temporal trends of surgical and interventional procedures of pulmonary valve implantation, clearly showing PPVI overtaking the number of surgical procedures from 2017 onwards.

### 4.4. RV-PA Conduit

**Figure 4:**
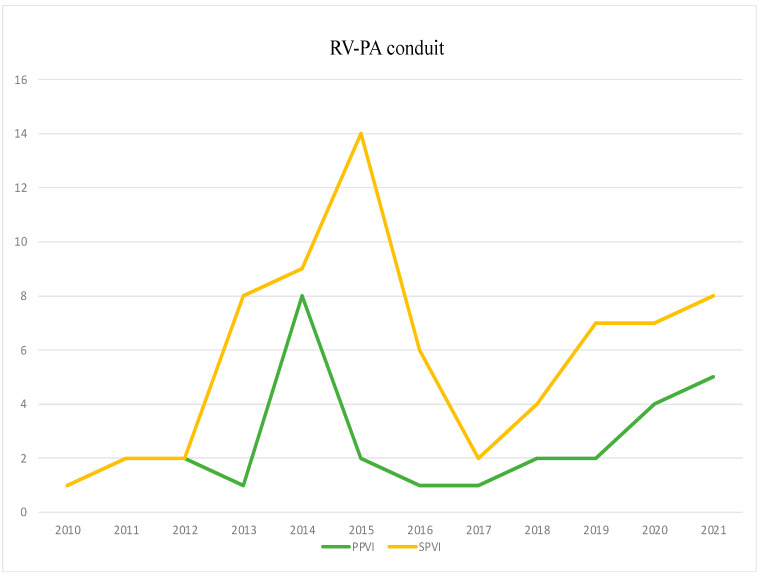
Trends in percutaneous (PPVI) and surgical (SPVI) pulmonary valve implantation in patients with right ventricle to pulmonary artery conduit.

The patients with RV-PA conduit included those with rToF, palliation for pulmonary atresia with ventricular septal defect (PA-VSD), aortic valve disease after a Ross procedure, and a Rastelli operation for the transposition of the great arteries. The number of diagnostic procedures did not differ between the two groups (47 vs. 48, *p* > 0.05), while the number of interventional catheterizations increased from 21 to 42 (*p* < 0.01), mainly due to PPVI procedures. Figure 4 shows the homogeneous temporal trend of PPVI with respect to SPVI in this subset of patients.

### 4.5. Aortic Coarctation

Among the patients with native or already operated aortic coarctation, there were no real differences between the two groups. There is a negative trend for diagnostic catheterizations, probably due to the increasing accuracy and reliability of non-invasive diagnostic tools (CT, MRI), which are now routinely used for the evaluation of patients with aortic arch obstruction.

### 4.6. Atrial Switch for TGA

Among a total of 63 patients who had Mustard or Senning repair of TGA, 84 procedures were performed over the entire study period. While there was no significant difference concerning diagnostic studies, most of these are due to concerns about pulmonary pressures, stenting of Mustard baffles became increasingly more common (Table 2).

### 4.7. Arterial Switch for TGA

The 63 patients who had arterial switch operation for TGA underwent 70 procedures. While it was common in the past to check for coronary and pulmonary arteries some time after repair in these patients, in recent years, CT and MRI scans took over cardiac catheterization as a diagnostic tool, thus leading to a significant decline in non-interventional invasive procedures (Table 2). On the contrary, the number of interventional procedures remained stable over time.

### 4.8. PA/IVS

Among 10 patients with pulmonary atresia with PA/IVS and biventricular repair, 22 procedures were carried out (Table 2). There was a significant increase in interventional procedures over time, mainly due to the need for pulmonary artery stenting. Atrial septal defect closure was performed in two patients only, one in each group.

## 5. Discussion

The reduction in mortality rate in patients with CHD has led to a shift of the epidemiology. Over the last decade, two-thirds of patients with CHD have been adults [2].

According to the Global Burden of Disease Study, from 1990 to 2017, there was a 18.8% increase in the prevalence of CHD [14]. Van Der Bom reported that 15% of cases of ACHD are moderate and 3% are severe [15].

The importance of a dedicated and highly specialized team was first introduced in 2000 during the 32nd Bethesda Conference [16]. Later, Cordina et al. reported adverse to catastrophic events if ACHD patients were referred to a general cardiologist [17]. These data are confirmed by Mylottie et al., who demonstrated (Quebec registry) a better survival rate when patients were referred to a dedicated center [18].

In our hospital, in 2016, we started a dedicated program and set up a multidisciplinary approach according to different subsets of ACHD patients. In this study, we sought to evaluate if this effort has changed the selection criteria and results for patients who are candidates for an invasive cardiological assessment in the catheterization laboratory.

In our case series, there has been an increase in the total number of diagnostic procedures, mostly in Fontan patients. This is the result of creating a dedicated Fontan clinic within the ACHD group, and the emerging need for a risk stratification of these complex patients, according to current guidelines [19].

In our experience, extracardiac Fontan conduit stenting is the kind of percutaneous intervention that had the most notable increase in the second time frame, while the numbers of other types of percutaneous treatment were stable in both periods. Indeed, during the first period, the absence of clear-cut guidelines led to heterogenous strategies, often denying the potential optimization of Fontan circulation just because a patient was considered stable and free of symptoms. Moreover, standard, invasive, diagnostic follow-up was previously deemed unnecessary by many clinical cardiologists. Concerning the closure of Fontan fenestrations, we observed that most patients were treated in the first period but experienced a progressive reduction over time. The long-term management of Fontan fenestrations is controversial in the literature and is based on retrospective observational studies [20]. This led to important differences in long-term fenestration management among different centers [20]. In our experience, fenestration closure has commonly been carried out in the past. In fact, as demonstrated by Ozawa et al. [21], in Fontan patients undergoing cardiac catheterizations after Fontan fenestration closure, the development of new venous–venous collaterals was observed, which act as new decompression pathways and cause increased desaturation.

As for post-Mustard-operation stenotic baffles, we observed a consistent increase in percutaneous treatment with or without the extraction of trapped leads. This treatment was administered using a multidisciplinary approach with electrophysiologists and cardiac surgeons.

Also, the number of PPVI procedures increased in the second period, but this was the result not only of the institution of the ACHD Unit, but also of the availability of new devices for treatment of the native outflow tract, as described in the literature [22]. In fact, new devices like the new self-expanding pulmonary valves are increasing the indications for percutaneous procedures even for large outflow tracts, until now reserved to cardiac surgery. In our tertiary center, the development of multimodality imaging—such as advanced cardiac imaging, MRI with fluid dynamics, multimodal integration, 3D print CT, and virtual 3D modelling—can ensure that DCCs are replaced with noninvasive tools in all patients, except for those with a Fontan. This approach reduces the rate of hospitalizations, radiation exposure, and complications associated with an invasive procedure.

These data and the establishment of a dedicated ACHD working group in a children’s hospital were possible due to the assembly of a multidisciplinary team with cardiologists that were highly skilled in congenital heart disease and pediatric cardiology, but at the same time had experience in adult cardiology. Furthermore, considering the comorbidities related to adulthood, and in anticipation of acquired cardiovascular diseases, our hospital is at the center of a network with tertiary hospitals for adults in the same city. In accordance with the global consensus statement of Moons et al. [23], our hospital offers shared management of patients with adult centers that have a multidisciplinary team that can address any type of comorbidity requirement. For example, patients with Fontan circulation who develop FALD (Fontan-Associated Liver Disease) undergo cardiological follow-up at our hospital and referred to an adult referral hospital for liver follow-up. The two teams of doctors compare and consult periodically to determine the best treatment for the patients. This approach is also used for other comorbidities: renal insufficiency, onco-hematological diseases, psychiatric diseases, and gynecological diseases. An important aspect is also pregnancy in women with ACHD. Again, the patient is closely followed up with a joint team of cardiologists from the children’s hospital and gynecologists from an adult referral hospital. At the end of the pregnancy, the patient gives birth in the adult hospital, always under the supervision of the multidisciplinary team. Also, for acquired cardiovascular diseases, such as dyslipidemia or atherosclerosis, or in the case of a heart transplant, there is a network of referral hospitals for adults with different specialist experience regarding patient needs. The team jointly decides where it is more appropriate to treat a patient in relation to their known comorbidities and the risk of onset.

## 6. Study Limitations

This study has some limitations. It is an observational retrospective study based on administrative and recorded data. Although from 2016 the number of DCCs and ICCs showed a fairly evident rise in the subset of patients with Fontan circulation, among the other patients, this could be less clear. The potential inference of unknown factors other than the creation of an ACHD unit in determining the observed data cannot be completely ruled out. However, given the demographic homogeneity of the entire study group, we are inclined to think that these changes are related to the impact of the establishment of our dedicated team, especially in Fontan patients.

## 7. Conclusions

To the best of our knowledge, this is the first study where moderate and severe CHD were considered for the selection of diagnostic or interventional cardiac catheterization before and after a dedicated program.

The patients who showed the highest impact were those with a Fontan operation who were followed up by a highly qualified multidisciplinary team, leading to a tailored treatment using a percutaneous approach.

Moreover, the diagnostic studies performed by an experienced operator can highlight hemodynamic status and stratify risks. This may avoid late referral for heart transplantation in selected patients.

## Figures and Tables

**Table 1:** Demographic and clinical data of the study population.

	Group A n (%)	Group B n (%)	*p* (Two-Paired *t*-Test)
N. of patients	194	400	
Mean age at first catheterization as ACHD patient (yrs.)	24 ± 6	27 ± 6	<0.01
Genetic syndrome	17	19	ns
Fontan palliation	61	152	<0.01
rToF outflow native	32	47	ns
RV-AP conduit	44	53	ns
TGA (atrial switch)	24	46	ns
TGA (arterial switch)	42	21	ns
PA/IVS	5	5	ns
CoAo	25	7	ns
CCTGA	3	1	ns
Ebstein anomaly	1	3	ns
AVSD	4	2	ns
Coronary anomalies	8	2	ns
Others	3	2	ns

RV-AP: right ventricle–pulmonary artery; rToF outflow native: repaired tetralogy of Fallot with native right ventricle outflow tract; CoAo: coarctation native or after surgery; TGA: transposition of great arteries; CCTGA: congenitally corrected transposition of great arteries; PA/IVS: pulmonary atresia with intact ventricular septum (two or one-half repair); AVSD: atrioventricular septal defect; ns: not significant.

**Table 2:** Distribution of diagnostic and interventional procedures among the two groups and in relation to native or post-surgical anatomy.

	Diagnostic Procedures (%)		*p*-*Value*	Interventional Procedures (%)		*p*-*Value*
	Group A	Group B	*p*	Group A	Group B	*p*
Overall	224 (13.6)	280 (16.9)	<0.01	124 (7.4)	211 (12.6)	<0.001
Fontan	54 (17.4)	151 (48.5)	<0.001	53 (17)	57 (18.3)	ns
RV-PA cond.	47 (21.4)	48 (22.7)	ns	21 (9.5)	42 (19.1)	0.003
rToF native	28 (7.6)	18 (4.9)	ns	24 (6.5)	46 (12.5)	0.006
CoAo	13 (5.8)	7 (3.6)	ns	11 (5.7)	13 (6.8)	ns
Arterial switch	40 (25.6)	15 (9.6)	<0.001	7 (4.5)	8 (5.1)	ns
Atrial switch	20 (20.8)	30 (31.2)	ns	6 (6.2)	28 (29.2)	<0.001
PA/IVS	8 (29.6)	3 (11.1)	ns	2 (4.2)	9 (18.7)	0.01
Ebstein native	2 (7.1)	1 (3.6)	ns	0 (0)	3 (10.7)	ns
Coronary bn	5 (19.2)	2 (7.7)	ns	0 (0)	2 (7.7)	ns
AVSD	5 (2.9)	2 (1.1)	ns	0 (0)	1 (0.6)	ns
Other	2 (28.5)	3 (42.8)	ns	0 (0)	2 (28.6)	ns

RV-AP cond.: right ventricle–pulmonary artery conduit; rToF native: repaired tetralogy of Fallot with native right ventricle outflow tract; CoAo: aortic coarctation native or after surgery; TGA: transposition of the great arteries; Ebstein native: Ebstein anomaly of the tricuspid valve; PA/IVS: intact septal pulmonary atresia; AVSD: atrioventricular septal defect; ns: not significant; *p*-values refer to difference between groups.

## Data Availability

The data presented in this study are available on request from the corresponding authors.
